# Understanding Social Exercise: Desire and Intention to Participate in Running Crews

**DOI:** 10.3390/ijerph20032371

**Published:** 2023-01-29

**Authors:** Doyeon Won, Hyung-hoon Kim, Jung-sup Bae

**Affiliations:** 1 Department of Kinesiology, Texas A&M University-Corpus Christi, Corpus Christi, TX 78412, USA; 2 Department of Taekwondo and Security, Honam University, Gwangju 62399, Republic of Korea; 3 College of Liberal Arts, Konkuk University, Chungju 27478, Republic of Korea

**Keywords:** social running, running crew, model of goal-directed behavior, health promotion

## Abstract

The current study investigated the determinants of the desire and intention to participate in an inner-city ‘running crew’ among social runners using a theoretical framework of the model of goal-directed behavior (MGB). Data were obtained from 245 social runners in Korea using an online questionnaire and primarily analyzed with the structural equation modeling technique. The results indicated that the desire to participate in a running crew was influenced most by positive anticipated emotions, followed (in descending order) by attitudes, negative anticipated emotions, social norms, perceived behavioral control, and the frequency of past behaviors. Runners’ behavioral intentions were predicted by desire but not directly related to perceived behavioral control and the frequency of past behavior. Overall, the study emphasized the importance of positive anticipated emotions and behavioral desire, among others, to encourage recreational runners’ participation in social running activities. Given that social runners rely on mobile apps to participate in running crew activities, the current study’s results have practical implications for running crew organizers, sports-branded app developers, and health promotion agencies.

## 1. Introduction

According to the Ministry of Culture, Sports, and Tourism of Korea, approximately 62.2% of the general public participates in physical activities (PAs) at least once a week, and 52.4% participate in ‘sport-for-all’ activities at least twice a week [1]. In addition, the average monthly expenditure on PA or sports activities increased from KRW 50,000 in 2016 to KRW 65,000 in 2018 [1,2]. Overall, people in Korea are investing a greater amount of their time and money in PA and sports as they pay more attention to their health and well-being [2]. Among various PA options, running is one of the popular activities across nations. For example, according to the American Time Use Survey, running was the fourth most popular activity during the 2009–2015 timeframe as, of those who engaged in sports or exercise, 8.6% of people in the U.S. engaged in running on the days they exercised [3]. Similarly, running was the fourth most frequently undertaken physical activity in England, preceded only by walking for leisure, walking for travel, and fitness activities, during the peak of the COVID-19 pandemic from mid-May 2020 to mid-May 2021 [4]. 

However, running is not well received by the general public in Korea [2], partly due to the risk of traffic-related injuries, other pedestrian safety concerns, and the possibility of encountering unpleasant people during the activity. In addition, one of the main issues in sports is continued participation in sport activities because of the current low retention rate of sports participation in Korea [2]. Relative to team- or group-based sports activities, such as basketball and soccer, Koreans tend to consider running as an individual sports activity; thus, running has not been socially consumed to a considerable extent. From a motivational perspective, participating in physical activity or exercising with others (i.e., social or group exercise) would be a much more effective way of encouraging individuals to participate in physical activities due to the opportunities for social engagement and interaction engendered by these pursuits, especially considering the advances in technology such as mobile apps and social media channels [5,6,7,8]. In particular, running is an example of a sports activity that is profoundly socially structured [7,8]. Thus, the number of co-runners, regardless of the co-runners’ ability level, is significantly associated with increases in running behavior (e.g., weekly running sessions). In addition, those who exercise with others are likely to acquire greater health benefits in comparison to those exercising alone [5]. Thus, social running could be an effective way of encouraging participation in running to promote health and psycho-social well-being. 

While the COVID-19-related restrictions on public gatherings and sport events have been lifted in many countries, some countries, including Korea, still have relatively strict policies and regulations on such activities. For example, many public and private sport clubs and facilities in Korea offer only limited sport programs (e.g., some sports programs are not offered or only limited sections per sport are offered, fewer users are allowed per program, etc.) and have strict user policies (e.g., vaccine mandates; social distancing; and indoor-masking mandates). Logically, outdoor running is one of the recommended PAs during the pandemic and post-pandemic era. However, during the COVID-19 pandemic, runners reported reduced running-related behavior in terms of distance, frequency, and intensity during the pandemic [9,10]. 

Given the psychosocial and health benefits of socially consumed sports acquired by participants [7,8], the current study investigated the determinants of social running activities using the model of the goal-directed behavior (MGB) perspective. The results from the current study will provide valuable information for health promotion agencies and running app companies regarding the determination of a way in which to encourage people to participate in social running. 

### 1.1. Model of Goal-Directed Behavior (MGB)

Developed from the theory of planned behavior (TPB) [11,12], the MGB postulates that attitudes, subjective norms (SN), and perceived behavioral control (PBC), as well as both positive anticipated emotions (PAE) and negative anticipated emotions (NAE), predict whether people want to engage in something (i.e., desire) [13,14,15,16]. Subsequently, desire mediates the influence of the MGB determinants (i.e., attitudes, subjective norms, and PBC) on intention, while PBC also directly influences intention. In addition, the frequency of past behavior (FPB) is a meaningful predictor of both behavioral desire and behavioral intention [13,14,15,16]. The TPB has been an effective theoretical framework for understanding human social behavior [12]. However, the TPB has some limitations regarding predictive validity, the assumption of rationality, and possible omissions of potentially critical factors such as emotions, past behavior, and other background factors [12,13,14]. Even though the TPB indirectly accounts for some of the factors and determinants not explicitly included in the theoretical model, Ajzen called for expanded research to advance our understanding of human behavior [12]. In response to such criticism, the MGB was proposed to broaden the TPB by including desire toward a behavior, anticipated positive and negative emotions, and past behavior [13,14]. 

In sum, the MGB is a more effective decision-making model that has been widely applied in order to understand various human behavior [16]. For example, in the context of outdoor sport participation in China, Kim et al. suggested that the MGB is an effective framework for predicting individual desire and intention with respect to sport participation [17]. They found that PAE was the strongest predictor of behavioral desire, followed by FPB, PBC, and NAE. Relative to NAE, PAE was extremely important with respect to increasing outdoor participants’ behavioral desire. Subsequently, the behavioral intention concerning outdoor sport participation was highly related to behavioral desire, followed by FPB and PBC. Regarding physical activity, Esposito et al. claimed that attitudes were the strongest determinant of the behavioral desire for physical activity, followed by PBC, subjective norms, and PAE [15]. Further, they suggested that the MGD is more effective in terms of predicting the desire for and behavioral intention to engage in physical activity. As Perugini and Bagozzi claimed [13,14], subsequent studies found that significantly greater variances in intentions and behaviors are explained by the MGD model in comparison to the TPB [15]. Accordingly, although the MGD antecedents that most influence behavioral desires will differ depending on the study context, the MGD is still one of the most effective theoretical frameworks for predicting behavioral desires and, consequently, behavioral intentions [13,14,15,16]. Even though the MGD has been used frequently in recent years, the vast majority of the research studies were conducted in travel and tourism, spectator sports, or sporting goods contexts. Surprisingly, only a limited number of studies have applied the MGD framework to participation in sport settings. Therefore, the MGD was adopted as the framework for the current study. 

### 1.2. MGB-Related Determinants of Desire to Participate in Social Running

The MGD framework suggests that a desire toward a given behavior mediates the relationship between MGD determinants and behavioral intentions [13]. Specifically, the factors that influence one’s desires include attitudes, PAE, NAE, SN, PBC, and FPB; goal-related desire fully mediates the relationships of attitudes, PAE, NAE, and SN with respect to behavioral intentions, while it partially mediates the relationships of PBC and FPB with respect to intentions [13,14,15,16]. By predicting the level of outdoor sport participation in China, Kim et al. found that PAE was the most critical determinant, along with FPB, PBC, and NAE, while attitudes and SN were not statistically significant antecedents to desire in their study [17]. While examining Vietnamese intense adventure behavior (e.g., caving, rock climbing, scuba diving, and trekking), Bui and Kiatkawsin found that PBC was the most significant predictor of the desire to engage in intense adventure tourism, followed by attitude and PAE. Similarly, bicycle travelers’ desire for bicycle tourism was well explained by PAE, SN, PBC, FPB, and attitude (in this order) [18]. Given the nature of bicycle tourism (e.g., interactions with the natural environment, social participation in bicycling, etc.), PAE and SN were especially significant in this context. In the context of daily physical activity (e.g., public sports clubs), PAE and PBC were two primary predictors of continued participation in public sports clubs in Korea, while FPB was a weak but significant determinant [19]. In a recent meta-analysis on tourism and hospitality, PAE was the most influential antecedent to the formation of goal-oriented desire, followed by attitudes, SN, PBC, and FPB. The set of MGB antecedents explained 70.9% of the variance in goal-oriented desire [16].

Based on the predictive power of the MGB [13,14,15,16,17,18], the desire to participate in social running can be adequately explained by MGB determinants. For example, potential or returning social runners are likely to have a greater intention to participate in social running activities if a runner has a favorable evaluation of social running (attitude), is surrounded by friends who support social running activities (SN), perceives a greater perceived ease of participating in the social running activity (PBC), anticipates positive emotional reactions towards the behavior (PAE), expects negative emotions when failing to participate (NAE), and has a habit of participating in social running activities (FPB). Especially given the societal nature of social running or running crews [7,8,20], it is expected that PAE and SN are particularly important factors. Based on this rationale, the following set of hypotheses was developed.

**Hypothesis** **1** **(H1).** *Attitude toward social running has a positive influence on runners’ goal-oriented desire to participate in social running.*

**Hypothesis** **2** **(H2).** *Subjective norms (SNs) concerning social running have a positive influence on the desire to participate in social running.*

**Hypothesis** **3** **(H3).** *Positive anticipated emotion (PAE) toward social running has a positive influence on the desire to participate in social running.*

**Hypothesis** **4** **(H4).** *Negative anticipated emotion (NAE) toward social running has a positive influence on the desire to participate in social running.*

**Hypothesis** **5** **(H5).** *Perceived behavioral control (PBC) concerning social running has a positive influence on the desire to participate in social running.*

**Hypothesis** **6** **(H6).** *The frequency of past behavior (FPB) with respect to social running has a positive influence on the desire to participate in social running.*

### 1.3. MGB Determinants of Behavioral Intention in Social Running

The MGB framework includes various determinants for the prediction of individual intentions and behaviors, including volitional (attitude and SN), non-volitional (PBC), affective (PAE and NAE), automatic (FPB), and motivational (desire) processes [13,14,15,16,17,18]. The MGB posits that goal-oriented desire is a key mediator between MGB determinants and behavioral intention, wherein the latter is the most effective predictor of future behavior [13]. As discussed previously, goal-oriented desire fully mediates the influence of the effects of attitude, SN, PAE, and NAE on behavioral intention, while it partially mediates the influence of PBC and FPB [13,14,15].

Kim et al. found that behavioral desire was the most influential factor that predicted the behavioral intention to participate in outdoor sports along with PBC, while FPB was positively but not significantly associated with intentions [17]. Similarly, Song et al. found that desire and PBC were powerful antecedents in predicting behavioral intentions but not FPB in terms of understanding the behavioral intentions of nature-based festival attendees [21]. However, Chiu and Cho’s meta-analysis of tourism revealed that FPB is a weak but significant predictor of behavioral intention [16]. The results of Meng and Han’s study on bicycle tourists corroborate the MGB’s theoretical proposition that FPB is a relatively weak but significant determinant of intentions, and that goal-oriented desire partially mediates the relationship between FPB and intentions [22]. 

**Figure 1:**
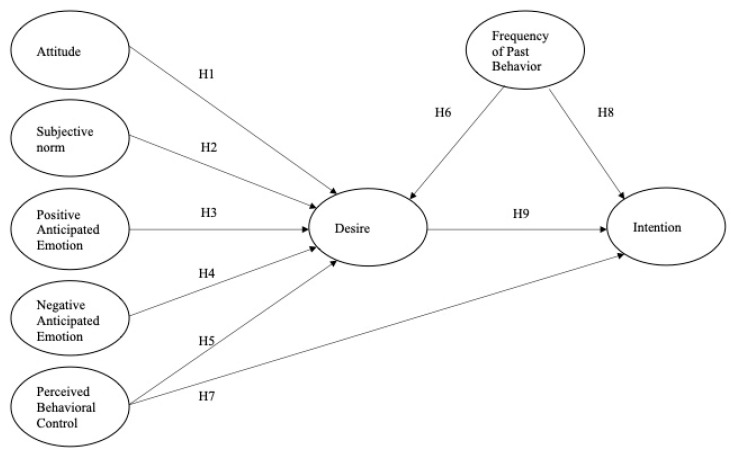
The conceptual model with study hypotheses.

In the social running context, potential and returning runners have high intention to participate in social running if they have a higher level of goal-oriented desire (motivational process); think of themselves as having the resources, abilities, and opportunities to participate in social running with ease (PBC); and are familiar with social running and have a habit of running as a social group (FPB). Thus, the following hypotheses were presented. The research model of the current study is summarized in Figure 1.

**Hypothesis** **7** **(H7).** *PBC concerning social running has a positive influence on the intention to participate in social running.*

**Hypothesis** **8** **(H8).** *FPB concerning social running has a positive influence on the intention to participate in social running.*

**Hypothesis** **9** **(H9).** *Desire concerning social running has a positive influence on the intention to participate in social running.*

## 2. Materials and Methods

### 2.1. Participants and Procedures

For the current study, data were collected from 250 running crew members who participated in social running activities in Seoul, Korea, using a convenience-sampling method. An online survey link was sent to 10 running crew groups via social media sites (e.g., Instagram) to collect data. The participants voluntarily responded to the survey as there was no financial incentive to return this survey. 

After removing five incomplete or invalid responses, a total of 245 completed questionnaires were considered for analysis. Of the 245 respondents, 52.2% were males (n = 128) and 48.8% were females (n = 117). In terms of age, 28.5% were under 20 years of age (n = 65), 39.6% were aged between 21 and 30, 22.9% were aged between 31 and 40, and 11.0% were over 41 years old. The vast majority of the respondents participated in running crew activities more than five times per month (n = 214; 87.4%). About 26.9% (n = 64) had been part of a running crew for less than two years, 60.5% (n = 149) had been a member for more than two years but less than four years, and 12.2% (n = 30) had been a member for more than four years (see Table 1).

### 2.2. Instrument

The scales used to measure the MGB constructs were taken from relevant studies that investigated the role of MGB determinants with respect to predicting individuals’ behavioral intentions and behaviors [11,13,21]. The survey instrument comprised the following variables, including attitudes towards the running crew (4 items), subjective norms (4 items), perceived behavioral control (PBC; 4 items), positive anticipated emotions (4 items), negative anticipated emotions (4 items), desire (3 items), and behavioral intention (4 items), as well as demographic variables such as age, gender, and running crew-related questions (e.g., the frequency of past behavior; FPB). 

All items, excluding demographic variables, were measured using a five-point Likert-type scale ranging from 1 (strongly disagree) to 5 (strongly agree). Some illustrative examples of the scale items are as follows: “I think that for me to participate in a running crew is enjoyable” (attitude), “most of the other people important to me think I should participate in running crews” (subjective norms), “If I wanted to, it would be easy for me to participate in a running crew” (PBC), “If I can participate in a running crew during the next week, I will feel excited” (PAE), “If I cannot participate in a running crew during the next week, I will feel disappointed” (NAE), “I am eager to participate in a running crew” (Desire), and “I am planning to participate in running crews in the near future” (intention). See Table 1 for the constructs and items used in this study. 

### 2.3. Statistical Analysis

Structural equation modeling (SEM) was used to test a set of study hypotheses for the current study. The normality assumption of the data was examined first; consequently, it was determined that there were no such issues. Data were then analyzed using the two-step modeling approach of SEM [23,24]. First, the measurement model was tested using a confirmatory factor analysis (CFA) to establish the construct reliability and validity. Subsequently, the structural model was tested to examine the relationships between the study variables. 

## 3. Results

### 3.1. Measurement Model’s Assessment

To assess the psychometric properties of the measurement model, CFA was conducted [25]. The results showed that the measurement model fitted the data adequately: χ^2^ (303) = 686.78, χ^2^/df = 2.27, CFI = 0.93, TLI = 0.92, and RMSEA = 0.07. Table 2 provides the reliability and validity scores of the study variables, including correlations between the study variables, Cronbach’s alpha values, composite reliability scores, and the average variance explained (AVE) as well as the means and standard deviations (*S.D*.) for the variables used in this study [26,27,28].

All the correlations between the study variables were well below 0.85; thus, there were no major multicollinearity issues. The highest correlation was found between desire and intention (*r* = 0.56; *p <* 0.001). In addition, desire was significantly correlated with SN (*r* = 0.53; *p < 0*.001), PAE (*r* = 0.39; *p* < 0.001), FPB (*r* = 0.32; *p* < 0.001), PBC (*r* = 0.30; *p* < 0.001), NAE (*r* = 0.24; *p* < 0.001), and attitude (*r* = 0.16; *p* < 0.05), and according to this order. Intention was weakly but significantly correlated with FPB (*r* = 0.31, *p* < 0.001) and PBC (*r* = 0.22, *p* < 0.01). 

Cronbach’s alpha coefficients and composite reliability (CR) values were used to evaluate the reliability of the measures used in this study [26,27,28]. The results indicated that the scale demonstrated good reliability given that the Cronbach’s alpha values of all constructs were above the suggested threshold of 0.70, ranging from 0.79 to 0.95, and all the CR values were above the threshold criterion of 0.70, ranging from 0.86 to 0.98. 

As reported in Table 2, the construct and convergent validity of the measures were assessed by evaluating the factor loadings and average variance extracted (AVE) values. All the factor loadings of the measures were highly significant, ranging from 0.62 to 0.94 (*p* < 0.001). The AVE values ranged from 0.61 to 0.92 and thus fulfilled the threshold criteria of 0.50. In brief, the measurement model exhibited adequate psychometric properties (see Table 2). 

### 3.2. Structural Model Assessment

**Figure 2:**
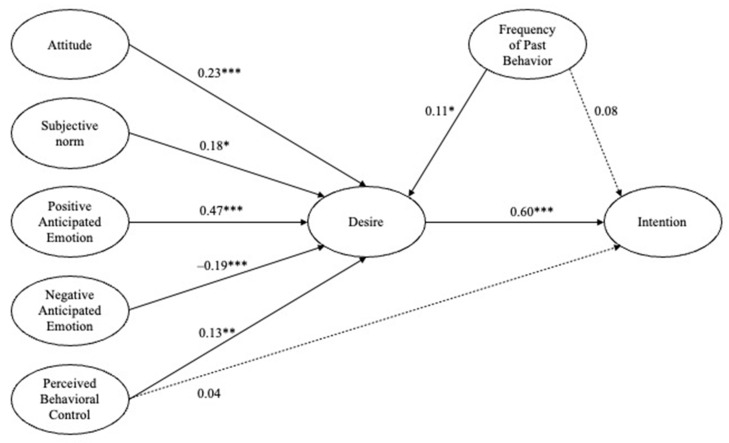
The research model with path coefficients. * *p* < 0.05; ** *p* < 0.01; *** *p* < 0.001.

SEM was used to analyze the relationships between the study variables. Prior to evaluating the structural model, multicollinearity between the endogenous variables was assessed using variance inflation factor (VIF) values [29]. All the VIF values were lower than the threshold value of 5.0, suggesting no significant collinearity issue. As reported in Table 3, the results of the SEM showed that the proposed structural model fitted the data reasonably well: χ^2^ (332) = 770.19, χ^2^/df = 2.32, CFI = 0.92, TLI = 0.91, and RMSEA = 0.07. Table 3 and Figure 2 report the results of the hypotheses tested in this study. 

The hypotheses in this study were tested using standardized regression coefficients of each path. Of the six MGB determinants of the desire to participate in a running crew, PAE was the most critical predictor of this desire (*β* = 0.47, *p* < 0.001), followed by attitudes (*β* = 0.23, *p* < 0.001), NAE (*β* = –0.19, *p* < 0.001), SN (*β* = 0.18, *p* < 0.05), PBC (*β* = 0.13, *p* < 0.01), and FPB (*β* = 0.11, *p* < 0.05), thus supporting H1, H2, H3, H4, H4, H5, and H6. All six MGB determinants were directly associated with runners’ desires to participate in a running crew. 

In addition, the participants’ behavioral intentions were strongly associated with desire (*β* = 0.60, *p* < 0.001) but not directly related to PBC and FPB, thus supporting H9 but rejecting H7 and H8. Overall, the current study suggests a full-mediatory role of behavioral desires in the relationship between the MGB determinants and intentions in this study’s context.

### 3.3. Multigroup Analyses

To control the potential influence of background characteristics, a series of t-tests and ANOVAs were performed to investigate the moderating roles of age and gender. For ease of calculation and analysis, age was divided into two groups (under 30 years old = 162; over 31 years old = 83). Consequently, no statistical group differences in terms of mean scores were found, except for a gender difference on NAE (*p* = 0.047). 

Even though we did not find major group differences based on the respondents’ gender and age, subsequent multigroup SEM analyses guided by the previous literature [8] were performed to explore the gender differences in this research model. The multigroup SEM across gender compared the unconstrained and constrained models [23]. The value of the chi-square differences (Δχ^2^) between the two models was insignificant, Δχ^2^ = 2.75 (df = 9) and *p* = 0.973, indicating that no moderating effects of gender exist in this research model. Similar to the multigroup SEM applied across gender, the analysis based on age groups found no significant differences between the unconstrained and constrained models (Δχ^2^ = 12.01 (df = 9); *p* = 0.213). These results suggested a configural invariance, and the two groups are equivalent. 

## 4. Discussion

Drawing from the MGB framework [13,14,15], the purpose of the current study was to examine the factors that influence the decision-making process of running crew participants. The current study found that all MGB determinants included in this study were significantly associated with the running crew’s desires, while positive anticipated emotions were the most influential determinant among all the other determinants. Meanwhile, the participants’ intention to participate in running crew activities was predicted by desire but not by perceived behavioral control (PBC) and the frequency of past behavior (FPB), contrary to the theory’s prediction. 

Firstly, the likelihood of participation in running crew activities increases as the level of desire increases, which is affected by positive anticipated emotions (PAE), attitudes toward social running activities (Attitude), negative anticipated emotions (NAE), subjective norms concerning running crew activities (SN), PBC, and FPB (in that order). The results suggest that in the running crew context (i.e., social sports consumption), where sustainable participation is a primary goal to enhance physical fitness and socio-psychological well-being, (potential) runners are likely to have a strong(er) desire to participate in running crew activities when they find both utilitarian and hedonic benefits in the activity and positively evaluate their situation and self-efficacy. Therefore, health promotion agencies, social running organizers, and running app developers should prioritize the development of strategies to enrich runners’ (or app users’) desire, for example, by enhancing runners’ anticipated emotions and promoting the benefits of social running activities. More detailed methods for fostering runners’ behavioral desires are discussed below. 

The current study found that PAE was the most influential factor that influences recreational runners’ desire to participate in running crew activities. The MGB theory posits that individuals’ desire to engage in a particular behavior is influenced by various processes, such as volitional (attitude and SN), non-volitional (PBC), affective (PAE and NAE), and automatic (FPB) processes [13,14,15,16,17,18]. Esposito et al. claimed that attitudes were the strongest determinant of behavioral desire for physical activity, followed by PBC, subjective norms, and PAE [15]. However, in the context of social running activities, the results of the current study suggest that the affective factor (e.g., PAE) might be the most influential determinant for runners’ desire to (continue to) participate in social running activities. This finding can be corroborated by two recent studies regarding outdoor sport participation and bicycle tourism in China [17,22]. 

Running in a social running crew can be considered both a functional (e.g., by enhancing health) and hedonic (e.g., by meeting new friends) activity. However, given the nature of outdoor running (i.e., individuals can run outside by themselves without the presence of others), outdoor runners may be inclined towards more hedonic-oriented consumption processes when deciding whether to participate in a social running activity. Consequently, the predictive power of PAE is relatively more important in this situation compared to non-social running contexts. Ekkekakis and Zenko argued that the traditionally predominant cognitivist approach (i.e., the utilitarian paradigm) within exercise psychology had overlooked the contribution of non-rational processes (i.e., the hedonic paradigm) to decision making with respect to exercise behavior, resulting in a lack of progress in raising the rates of physical activity and exercise at the population level [30]. Gardner et al. claimed that sport participants’ levels of perceived enjoyment are among the most critical factors influencing their continued participation in organized sports [31]. Therefore, from a health communication perspective, social running organizers and app developers should promote the hedonic benefits of running or exercising in a social context as opposed to over-emphasizing the utilitarian benefits of such activities. Gamification features should be designed to influence runners’ affective processes with respect to decision making [32]. 

The volitional factors (i.e., attitude and SN) were significant determinants of desire in this study [11,12,13,14]. However, the relative strength of the volitional factors was weaker than that of the affective factors (e.g., PAE). Attitudes towards and subjective norms concerning social running (e.g., a running crew) were meaningful antecedents to desire, and they also indirectly predicted the runners’ social running intentions, highlighting the importance of attitude formation (and attitude change) and peer influences concerning social running activities. Typically, outdoor running is a desirable activity from a health promotion perspective, but people have mixed attitudes towards social and/or outdoor running in inner-city settings due to the risk of potential infection, traffic-related injuries, other pedestrian safety concerns, and the possibility of encountering unpleasant people while engaging in said activity. Family and peer influence can prevent runners from participating in outdoor social running activities if people around the runner have negative attitudes toward the activity. From a different angle, (potential) runners are likely to have a stronger behavioral desire and intention toward social running if surrounded by those with positive attitudes or experiences concerning social running. Health promotion agencies, social running organizers, and app developers should provide more information about safer running routes and injury prevention tips for participants. Running clubs should establish a strict code of conduct and enforce this code to mitigate between-participant issues. 

Lastly, the findings suggest that non-volitional (i.e., PBC) and habitual (i.e., FPB) factors also influence runners’ desire to participate in social running activities. Bui and Kiatkawsin found that PBC was the most significant predictor of the desire to engage in intense adventure tourism [18]. However, in the social running context, PBC was a significant but relatively less critical factor influencing social runners’ desires. Runners are likely to have a stronger desire to re-participate if they positively evaluate their situation and have a habit of engaging in social running. As mentioned above, running-related information provided by running clubs, app developers, and health promotion agencies will be useful in mitigating constraints concerning running (e.g., lack of information, safety concerns, etc.) and thus facilitating participation and regular engagement in running as a habit. 

Despite the valuable insights from the current study’s findings, this study has some limitations. This study included running app users in the largest metropolitan city in Korea. Thus, the findings of this study may not be generalized to other cities and countries (with significantly different characteristics in terms of, e.g., population density). In addition, the data for the current study were collected from those who have some interest or experience in social running (i.e., a follower of a running club’s social networking sites or a member of a social running crew); thus, they might have a relatively higher level of motivation toward participating in running crews. From the self-determination perspective, individual motivation, namely, intrinsic motivation, extrinsic motivation, and amotivation, could be used to derive runners’ behavior in certain ways [7,33]. Unfortunately, the current study did not explore how different motivations would influence their participation in social running. However, the MGB posits that desires “transfer the motivational content to act embedded in attitudes towards the act, anticipated emotions, subjective norms, and PBC” [12] (p. 80). Thus, social runners’ motivations are at least indirectly reflected in the MGB. Nonetheless, future studies might consider expanding the MGB by including motivational factors to enhance the predictive validity of the MGB. In addition, the current study focused on understanding the continued participation in social running activities because sustained participation in sports is a critical issue for sports agencies in Korea [2]. Consequently, the findings of the current study should be interpreted with caution when applying this study to less-social and less-motivated sport participants. 

Relatively younger participants (i.e., under the age of 40) were included in this study. This might have led to age-related bias and limited the possibility of comparing the age differences (e.g., in comparison to the elderly) with respect to determining the factors that influence participants’ desire to engage in social running. In this study, there was no age-based difference that influenced the analysis of social running behavior. However, this might be related to the sample’s age profiles and data collection method (i.e., collected via social media). It is logical to think that younger runners are more likely to participate in running sessions and events more frequently than their older counterparts and have a larger number of co-runners (i.e., a bigger core sports network) [7,8]. According to a study that explored the Japan Gerontological Evaluation Study (JAGES) Cohort Study data, exercising alone and exercising with others both seem to have health benefits, although the increased frequency of exercise with others has additional health benefits to the elderly populations in Japan [34]. Even though, from an MGB perspective, the factors that influence the elderly’s desire and intention to engage in social running or social exercise would be similar to other age groups, it will be worthwhile to investigate their goal-directed mechanisms given the profound health benefits that ‘exercising with others’ can be bestowed unto elderly populations. 

The current study did not find any gender-based differences. In addition, the current study did not collect additional individual information, such as educational background, which might be closely related to individual motivation, attitude, and subjective norms concerning social exercise [2,4,8]. However, background variables are known to influence individual social behavior, including sport-related behaviors [7,8,12]. Even though the current study found no gender-based differences, future studies must consider the potential influences of background variables, such as gender, household income, and educational attainment, on the analysis of the social consumption of sports. In addition, it is possible that cultural factors (e.g., social norms, subgroup cultures, or ethnic differences) related to social exercise might have played a role in this study. Relatedly, this study was conducted with a cross-sectional survey design. Thus, future studies may consider different data collection strategies, target populations, and research designs to uncover more information about social running activities. 

## 5. Conclusions

Outdoor running is one of the most popular and recommended physical activities; the popularity of running, in general, has decreased in the pandemic era, and it still has not returned to pre-pandemic levels [31]. Despite the pandemic, running in a social group (i.e., social running) is suggested as an effective way to boost participation in running and improve both the physical and socio-psychological well-being of the participants [7,8]. One of the main issues regarding sports participation in Korea is the low level of sustained and continued participation in sport activities (i.e., high dropout rates) [2]. The results of the present study suggest that affective and emotional factors such as PAE were the most important determinants with respect to participants’ desires to engage in social running activities, while all other MGB antecedents influenced their behavioral desires. Thus, health promotion agencies, social running organizers, and app developers (or sports brands and the designers of their running apps such as Nike and Nike Run+) should create an enjoyable environment for (potential) runners and communicate with them with regard to both the hedonic and utilitarian benefits of social running activities [35]. 

## Figures and Tables

**Table 1:** Constructs and items used in this study.

Constructs/Items
Attitude I think that participating in a running crew is enjoyable. I think that participating in a running crew is wise. I think that participating in a running crew is good. I think that participating in a running crew is beneficial.
Subjective norms Most of my friends think I should participate in a running crew. Most other people important to me think I should participate in a running crew. Most of my close family members think I should participate in a running crew. Most people around me support my participation in a running crew.
PBC If I wanted to, it would be easy for me to participate in a running crew. It is mostly up to me whether I participate in a running crew activity. I am capable of participating in a running crew activity. I have enough resources and opportunities to participate in a running crew activity.
PAE If I can participate in a running crew, I will be excited. If I can participate in a running crew, I will be proud. If I can participate in a running crew, I will be satisfied. If I can participate in a running crew, I will be happy.
NAE If I cannot participate in a running crew, I will be disappointed. If I cannot participate in a running crew, I will be unhappy. If I cannot participate in a running crew, I will be sad. If I cannot participate in a running crew, I will be angry.
Desire I am eager to participate in a running crew. I want to participate in a running crew. I hope to participate in a running crew.
Behavioral Intention I am willing to participate in a running crew in the near future. I will make an effort to participate in a running crew in the future. I intend to participate in a running crew in the future. I am planning to participate in a running crew (activity) in the future.

**Table 2:** Summary results of correlation, reliability, and validity analyses.

Variable	1	2	3	4	5	6	7	8
Attitude	1							
2. SN	0.23 ***	1						
3. PAE	0.09	0.36 ***	1					
4. NAE	0.18 **	0.46 ***	0.17 **	1				
5. PBC	0.13 *	0.20 **	0.12	0.03	1			
6. Desire	0.16 *	0.53 ***	0.39 ***	0.24 ***	0.30 ***	1		
7. Intention	0.15 *	0.42 ***	0.30 ***	0.24 ***	0.22 **	0.56 ***	1	
8. FPB	0.15 *	0.27 ***	0.23 **	0.16 *	0.26 **	0.32 ***	0.31 ***	1
Mean	4.52	4.55	4.53	4.21	3.14	4.31	4.39	3.66
S.D.	1.79	0.56	1.32	0.68	1.23	0.64	0.63	0.94
Cronbach’s α	0.79	0.93	0.87	0.94	0.91	0.88	0.95	-
AVE	0.61	0.90	0.78	0.77	0.64	0.83	0.92	-
CR	0.86	0.97	0.93	0.93	0.88	0.94	0.98	-

Note: SN = Subject norm; PBC = Perceived behavioral control; PAE = Positive anticipated emotions; NAE = Negative anticipated emotions; FPB = Frequency of past behavior; S.D. = Standard deviation; AVE = Average variance explained; CR = Composite reliability. * *p* < 0.05; ** *p* < 0.01; *** *p* < 0.001.

**Table 3:** Summary of hypothesis testing results.

Hypothesis	Path	Standardized Coefficient (β)	t-Value
H1	Attitude --> Desire	0.23	3.52 ***
H2	SN --> Desire	0.18	2.29 *
H3	PAE --> Desire	0.47	5.58 ***
H4	NAE --> Desire	–0.19	–4.06 ***
H5	PBC --> Desire	0.13	2.60 **
H6	FPB --> Desire	0.11	2.54 *
H7	PBC --> Intention	0.04	0.60
H8	FPB --> Intention	0.08	1.52
H9	Desire --> Intention	0.60	8.73 ***
Model fit: χ^2^ (332) = 770.19, χ^2^/df =2.32, CFI = 0.92, TLI = 0.91, RMSEA = 0.07			

Note: SN = Subject norm; PBC = Perceived behavioral control; PAE = Positive anticipated emotions; NAE = Negative anticipated emotions; FPB = Frequency of past behavior. * *p* < 0.05; ** *p* < 0.01; *** *p* < 0.001.

## Data Availability

The data that support the findings of this study are available on reasonable request from the corresponding author at khh@honam.ac.kr.
